# Endoscopic fenestration of a giant frontal arachnoid cyst: Operative technique and anatomical nuances

**DOI:** 10.1002/ccr3.5386

**Published:** 2022-03-17

**Authors:** Ryan Dimentberg, Austin J. Borja, Gregory Glauser, Steven Brem, Omar A. Choudhri

**Affiliations:** ^1^ Department of Neurosurgery University of Pennsylvania Philadelphia Pennsylvania USA

**Keywords:** cerebrovascular, endoscopic fenestration, giant frontal arachnoid cyst, stereotactic navigation

## Abstract

Endoscopic fenestration is best as it is minimally invasive and does not require hardware in the surgical site (Figure 1). This case shows the safety of endoscopic fenestration and the utility of operative adjuncts (*J Korean Med Sci*. 1999;14:443; *Neurosurg Focus*. 2005;19:E7).

## Graphical abstract

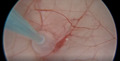

What are the key benefits to an endoscopic fenestration for the treatment of a giant arachnoid cyst causing mass effect? Endoscopic fenestration is minimally invasive and does not require hardware in the surgical site (Figure 1). This case shows the safety of endoscopic fenestration and the utility of operative adjuncts (Supplementary Video).^1^, ^2^

**FIGURE 1:**
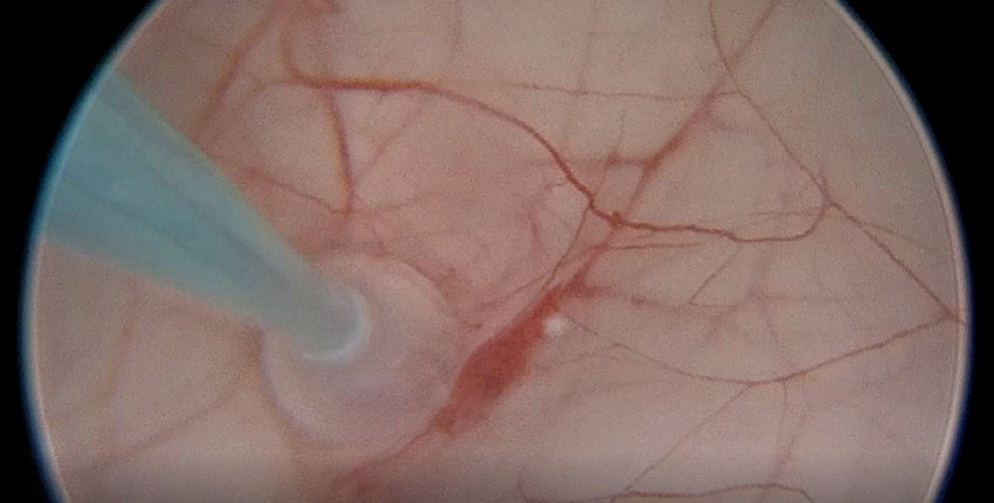
Intraoperative view showing fenestration of the giant left frontal arachnoid cyst

## CONFLICT OF INTEREST

The authors have no personal, financial, or institutional interest in any of the materials or devices described in the video.

## AUTHOR CONTRIBUTIONS

All authors involved in preparation of the video concur that no work resembling the enclosed video has been published or is being submitted for publication elsewhere. We certify that we have each made a substantial contribution as to qualify for authorship as follows: OAC and SB performed the procedure. OAC provided video narration. RD, AJB, and GG performed critical video editing and preparation for publication.

## ETHICAL APPROVAL

Strict adherence to all university, state, and federal requirements regarding patient confidentiality and care has been upheld.

## CONSENT

Written informed consent was obtained from the patient to publish this report in accordance with the journal's patient consent policy.

## Supporting information

Supplementary Material

## Data Availability

Data sharing not applicable to this article as no datasets were generated or analysed during the current study.
